# Addressing Tobacco in Managed Care: Results of the 2002 Survey

**Published:** 2004-09-15

**Authors:** Carol McPhillips-Tangum, Carmella Bocchino, Rita Carreon, Caroline Erceg, Bob Rehm

**Affiliations:** CMT Consulting; America’s Health Insurance Plans (AHIP), Washington, DC; America’s Health Insurance Plans (AHIP), Washington, DC; America’s Health Insurance Plans (AHIP), Washington, DC; America’s Health Insurance Plans (AHIP), Washington, DC

## Abstract

**Introduction:**

In the United States, tobacco use is the leading preventable cause of death and disease. The health and cost consequences of tobacco dependence have made treatment and prevention of tobacco use a key priority among multiple stakeholders, including health plans, insurers, providers, employers, and policymakers. In 2002, the third survey of tobacco control practices and policies in health plans was conducted by America's Health Insurance Plans' technical assistance office as part of the Addressing Tobacco in Managed Care (ATMC) program.

**Methods:**

The ATMC survey was conducted in the spring of 2002 via mail, e-mail, and fax. A 19-item survey instrument was developed and pilot-tested. Of the 19 items, 12 were the same as in previous years, four were modified to collect more detailed data on areas of key interest, and three were added to gain information about strategies to promote smoking cessation. The sample for the survey was drawn from the 687 plans listed in the national directory of member and nonmember health plans in America's Health Insurance Plans.

**Results:**

Of the 246 plans in the sample, 152 plans (62%) representing more than 43.5 million health maintenance organization members completed the survey. Results show that health plans are using evidence-based programs and clinical guidelines to address tobacco use. Compared to ATMC survey data collected in 1997 and 2000, the 2002 ATMC survey results indicate that more health plans are providing full coverage for first-line pharmacotherapies and telephone counseling for smoking cessation. Plans have also shown improvement in their ability to identify at least some members who smoke. Similarly, a greater percentage of plans are employing strategies to address smoking cessation during the postpartum period to prevent smoking relapse and during pediatric visits to reduce or eliminate children's exposure to environmental tobacco smoke.

**Conclusion:**

The results of the 2002 ATMC survey reflect both tremendous accomplishments and important opportunities for health plans to collaborate in tobacco control efforts. With appropriate support, analytical tools, and resources, it is likely that health plans, clinicians, providers, and consumers will continue to evolve in their efforts to reduce the negative consequences of tobacco use.

## Introduction

In the United States, tobacco use is the leading preventable cause of death and disease. Smoking kills more than 440,000 people in the United States each year, with most deaths occurring from lung cancer, ischemic heart disease, and chronic airway obstruction (1). Yet approximately 23% of American adults continue to smoke cigarettes (2). In 2000, it was estimated that approximately 8.6 million persons in the United States were living with at least one condition attributed to smoking (3).

The health consequences of tobacco use are accompanied by a staggering economic burden. Smoking caused more than $157 billion in annual health-related economic losses between 1995 and 1999, including $81.9 billion in smoking-related productivity losses and $75.5 billion in excess medical expenditures (1). Smoking-attributable neonatal expenditures were estimated at $366 million in 1996, or $704 per maternal smoker (1). Together, the consequences and costs of tobacco dependence have made treatment and prevention of tobacco use a key priority among multiple stakeholders, including health plans, insurers, providers, employers, and policymakers.

In 1997, The Robert Wood Johnson Foundation established a collaborative program, Addressing Tobacco in Managed Care (ATMC). This program is based on the understanding that health plans' comprehensive benefits, sophisticated information systems, and defined populations, as well as their ongoing partnerships with health care providers, are well suited to implement, evaluate, and sustain tobacco control interventions. ATMC includes a National Program Office based at the University of Wisconsin Medical School's Center for Tobacco Research and Intervention, and a national technical assistance office (NTAO) managed by America's Health Insurance Plans (AHIP), formerly known as the American Association of Health Plans (AAHP). The mission of the NTAO is to advance the integration of tobacco cessation and prevention programs into routine health care by increasing the number and quality of tobacco control initiatives within health plans.

The NTAO provides resources to health plans and insurers striving to develop tobacco control programs; conducts a benchmarking awards program to highlight exemplary health plan tobacco control initiatives; promotes best practices and partnerships through national conferences; and oversees the development of a business case model for smoking cessation. The NTAO has also conducted three surveys of health plans over the past six years to assess practices and policies related to tobacco control.

The ATMC baseline survey was conducted in 1997, followed by a similar survey in 2000. The results of both surveys were published in peer-reviewed journals in 1998 and 2002 (4,5). The purpose of this paper is to present the results of the 2002 ATMC survey; highlight changes from 1997 to 2002; cross-reference the findings with national guidelines and recommendations; and explore these findings and trends in light of the changing environment in which health plans operate and the public's attitude toward tobacco use.

## Methods

A 19-item survey instrument was developed and pilot-tested in the fall of 2001. The instrument was designed to assess new trends, barriers, and opportunities related to addressing tobacco control in health plans, identify new models or frameworks of care, and assess changes in health-plan–based tobacco control activities between 1997 and 2002. The sample for the survey was drawn from the 687 plans listed in AHIP's national directory of member and nonmember health plans. The directory was stratified based on health plan enrollment size, and a random sample of 246 health plans was selected. The sample size enables the detection of a 5% difference between proportions at α = .05 and β = .80.

The ATMC survey was conducted in the spring of 2002. As in 1997 and 2000, the 2002 survey was conducted via mail, e-mail, and fax, with telephone follow-up with nonrespondents at two, four, and six weeks after initial contact. The sample included large national plans that have local plans in multiple states. As in previous years, the corporate office of each national plan was asked to review the questionnaire and determine whether they would respond on behalf of their local plans or ask local plans to complete the questionnaires individually. Three of four national plans opted to respond on behalf of their local plans and their responses reflect 64% (97/152) of the responses.

The 2002 survey questionnaire was similar to the 2000 survey. Of the 19 items in the 2002 questionnaire, 12 were the same as in previous years, four were modified to collect more detailed data on areas of key interest (i.e., pharmaceutical coverage and system-level interventions), and three were added to gain information about strategies to promote smoking cessation. Based on feedback provided during pretesting, the majority of survey questions focused on smoking cessation despite recognition that tobacco cessation or tobacco control is a more encompassing term. Although we recognize that the preferred provider organization (PPO) product has grown in popularity, the 2002 ATMC survey asked respondents to answer all questions based on their best-selling commercial health maintenance organization (HMO) product to preserve the ability to make comparisons with previous years.

All analyses were performed with SPSS software (SPSS, Inc, Chicago, Ill). Chi-square tests and *t*-tests were used for comparisons, and results of these tests were considered statistically significant when the corresponding *P* value was ⩽ .05. Consistent with previous years, the data are unweighted to best describe the policies and practices of health plans.

## Results

Of the 246 plans in the sample, 152 (62%) completed and returned the survey. Collectively, the 152 plans represent more than 43.5 million HMO members. Respondent plans were predominantly independent practice association, network, and mixed models. Fifty-one percent were for-profit and publicly held; 24% were for-profit and privately held; 23% were not-for-profit; and 2% were mutual companies. A comparative analysis of respondents and nonrespondents to the 2002 survey indicated that there were no significant differences in size, tax status, or predominant model type between respondents and nonrespondents.

Among plans that responded to the 2002 ATMC survey, 71% reported having written clinical guidelines for smoking cessation. The majority of plans reported having guidelines that had been internally developed by the plan; few plans reported using the 2000 U.S. Public Health Service *Clinical Practice Guideline on Tobacco Use and Dependence* or the 1996 Agency for Health Care Policy and Research (now the Agency for Healthcare Research and Quality [AHRQ]) *Practice Guideline on Tobacco Cessation* (Table 1).

Nearly three quarters of all plans indicated that they could identify at least some individual plan members who smoke (Table 1). Among those plans that reported being able to identify individual smokers, the most common data sources are health risk appraisals and telephone interviews. Only 6% of plans use enrollment data to identify individual smokers.

The vast majority of health plans that responded to the survey reported that they provide full coverage for at least one type of pharmacotherapy used for tobacco cessation (Table 1). Bupropion, in the form of Wellbutrin, was the most commonly covered pharmacotherapy. Only 11% of plans reported that provision of full coverage for tobacco cessation pharmacotherapies is dependent on enrollment in a counseling or cessation program.

Full coverage for at least one type of behavioral intervention used for tobacco cessation was reported by the vast majority of health plans (Table 1). Telephone counseling was the most commonly covered behavioral intervention, followed by face-to-face counseling and self-help materials.

Health plans reported having a variety of strategies to encourage members to stop smoking during times that might be considered important teachable moments. The majority of health plans reported having a specific strategy to address smoking cessation during pregnancy and during treatment for chronic illnesses (Table 1).

Plans reported that a variety of strategies are used with providers and their office staff to promote smoking cessation among plan members. The majority of plans reported offering provider education and offering prompts and reminders to providers (Table 1). Provider prompts and reminders were coupled with provider education by 44% of plans. Few plans reported offering incentives to providers and their staff to promote smoking cessation.

Health plans reported that they require providers to carry out a variety of assessments and activities related to smoking that are in accordance with the clinical model of the 5 As: Ask, Advise, Assess, Assist, and Arrange (6). (The 2002 ATMC survey was fielded before the development of Assess willingness to quit.) The majority of plans require providers to ask new patients about smoking status and include smoking status as a vital sign (ask about smoking status at every visit) (Table 1). Fewer plans reported requiring providers to carry out activities aimed at advising, assisting, and following up with patients trying to quit smoking.

Although health plans reported a variety of barriers that limit their ability to effectively address tobacco control, the most common barriers relate to resources (e.g., inadequate staff, funding, competing priorities) and system issues (e.g., poor data collection, reporting, record maintenance). Other barriers included lack of patient demand, lack of purchaser demand, and delayed economic return on investment.

Tobacco control activities used by larger health plans are different from those used by smaller plans (Table 2). Based on the enrollment distribution of health plans in our sample, we defined larger plans as those with more than 250,000 members and smaller plans as those with less than or equal to 250,000 members. Larger plans were more likely than smaller plans to have written clinical guidelines for smoking cessation *P* < .001) and to have a specific strategy to address smoking cessation during specific times, such as adolescence, pregnancy, postpartum visits, and hospitalization *P* ranged from < .001 to .02). Smaller plans, more likely to be staff and group-model plans, were more likely to be able to identify individual plan members who smoke *P* < .001) and provide full coverage for some prescription pharmacotherapies used for smoking cessation *P* ranged from < .001 to .02).

Although the ATMC survey instruments used in 1997, 2000, and 2002 were not identical, the majority of core questions on pharmacotherapies, behavioral health, and smoking cessation strategies remained unchanged. The percentage of plans that provide full coverage for any type of pharmacotherapy used for smoking cessation more than tripled from 1997 to 2002 (*P* < .001) (Table 3). The percentage of plans able to identify individual smokers also increased *P* < .001). More plans reported providing full coverage for telephone counseling *P *= .04) and face-to-face counseling *P *= .011) in 2002 compared with both previous surveys.

From 1997 to 2002, there were large increases in the percentage of plans with strategies to address relapse prevention during the postpartum period (*P* = .02) and smoking cessation during treatment for chronic illness *P *= .002) and following a heart attack *P* = .004) Table 3).

Health plan performance on measures related to requiring providers to adhere to four of the 5 As varied in both directions between 2000 and 2002 (Table 3). Although comparable data on these variables were not collected in 1997, the percentage of plans that require providers to ask new patients about smoking status (*P* = .02) and strongly advise all smokers to quit (*P* = .02) decreased from 2000 to 2002, and the percentage of plans that require providers to include smoking as a vital sign (i.e., ask about it at every visit) (*P* = .28) and assist smokers by referring them into appropriate treatment (*P *= .33) increased modestly.

## Discussion

The results of the 2002 ATMC survey indicate that health plans are using evidence-based programs and clinical guidelines to address tobacco use. Clinical guidelines detail the most effective options for helping patients to quit smoking, and using strategies recommended in clinical guidelines is associated with greater success in helping smokers to quit (6,7). Although a large percentage of health plans reported having written clinical guidelines for tobacco cessation, it is possible that even more plans address tobacco cessation within other clinical guidelines used for managing or treating conditions in which tobacco use is identified as a comorbidity or risk factor (e.g., heart disease, diabetes, asthma). It is also noteworthy that more than half of the plans reported adopting internally developed guidelines, as opposed to guidelines developed by federal agencies and expert panels such as the U.S. Public Health Service (USPHS) and AHRQ. However, it is possible that plans reviewed such guidelines and integrated many or all of the key components into their own guidelines.

Plans showed remarkable improvement in 2002, compared with previous years, in identifying individual plan members who smoke. The ability to identify smokers is an important indicator of a plan's ability to remind or prompt providers to discuss and/or advise patients about smoking cessation. Such provider reminders are considered an effective strategy for supporting smoking cessation and are recommended by the Task Force on Community Preventive Services (7). The survey question, however, assesses the percentage of plans that can identify any members who smoke (rather than all members who smoke), and the methods that plans report using to identify smokers are most likely to identify subgroups of smokers (i.e., those that respond to health risk appraisals or surveys). Indeed, the ability of health plans to identify smokers is contingent upon members actively providing information about their smoking status during some interaction with the health plan, whether during enrollment, through a survey, or via some other point of contact.

The number of health plans providing full coverage for any type of pharmacotherapy for tobacco cessation more than tripled in 2002, compared with previous years. In the 2002 ATMC survey, nearly nine out of 10 plans reported providing full coverage for at least one type of pharmacotherapy for tobacco cessation. Consistent with recommendations based on the effectiveness of various prescription and over-the-counter tobacco cessation first-line pharmacotherapies (6), the majority of plans reported providing full coverage for bupropion. The significant increase in the number of plans that provide full coverage for at least one type of pharmacotherapy related to tobacco cessation is well aligned with the growing body of literature indicating that reduced out-of-pocket cost is associated with greater use of tobacco cessation programs and services (8-12) and may lead to increased rates of cessation (10,11).

Consistent with literature citing the effectiveness of telephone counseling and that smokers are more likely to use telephone counseling than to participate in individual or group counseling sessions (13,14), approximately half of plans surveyed provide full coverage for telephone counseling. It is possible that even more smokers have access to telephone counseling through the availability of state-sponsored quit lines. Less than 25% of plans impose an annual or lifetime limit on coverage for tobacco cessation treatments, indicating widespread acceptance of the USPHS guideline recommending coverage for repeated, intensive tobacco dependence counseling and pharmacotherapy (6).

The results of the 2002 ATMC survey also suggest that plans are paying close attention to pregnancy and the postpartum period to assist women to quit smoking. The large percentage of plans reporting strategies to address smoking cessation during and after pregnancy to prevent relapse may reflect greater health plan awareness of research that has demonstrated the cost-effectiveness of offering smoking cessation programs to pregnant women (15).

Overall, our results indicate the greatest improvement in tobacco control activities is at the health plan level as opposed to the physician level. For example, more plans report providing full coverage for pharmacotherapies than report requiring providers to carry out activities in support of the 5 As. This may be because most health plans (especially those that are not staff-model HMOs) find changing physician behavior to be a challenge. Although more plans are beginning to experiment with performance feedback as a way to change physician behavior, prompts, reminders, and provider training are more common strategies.

Health plans continue to report that resource limitations, including insufficient staff and inadequate funding, are leading barriers to adequately addressing tobacco control. Health plans may benefit from developing a business case model that stresses the importance of tobacco cessation to purchasers and advocates for resources to implement and maintain evidence-based tobacco cessation programs. Research supported by the NTAO is underway to provide an estimated return on investment for smoking cessation interventions, based primarily on smoking-attributable costs for health plans.

The ATMC survey and its findings have limitations. The response rate of approximately 60% is respectable, but leaves open the possibility of selection bias. Even though no significant differences were detected between respondents and nonrespondents on three key characteristics (size, tax status, predominant model type), respondents possibly differed from nonrespondents in ways that were not measured. Another limitation to the ATMC survey is that the psychometric properties of the questionnaire were not tested to assess reliability or validity. However, the survey design process did include substantial pretesting to increase the probability of including questions that were reliable and likely to yield valid responses. Additionally, we identified a potential limitation of the 1997 survey — it did not include a frame of reference for product type (e.g., HMO, PPO). When the survey does not specify product type, respondents tend to answer for the HMO product. Respondents were explicitly asked to answer for the HMO product in 2000 and 2002. However, the possibility remains that the change in frame of reference contributes to some differences in survey findings from 1997 to 2000 or 2002 (but not from 2000 to 2002).

Aside from the ATMC surveys, few surveys have assessed tobacco control practices and policies of health plans. Some surveys have focused on plans operating in a single state (9,16), some have included a narrow subset of plans (i.e., well-established nonprofit plans with a history of offering tobacco cessation programs) (17), and others have collected information about subsets of smokers within a plan (i.e., pregnant women) (18,19). Nevertheless, a 1999 survey of California health plans reported results comparable to our results: 85% of HMOs in the California survey covered at least one form of pharmacotherapy; 77% covered bupropion; 46% covered telephone counseling; and 54% covered individual counseling (16). However, the limited availability of comparable data prohibits comparisons of our findings with other surveys and underscores the importance of ATMC data for an adequate understanding of health plan tobacco control practices and policies at the national level.

The results of the 2002 ATMC survey indicate that an increasing number of health plans are using evidence-based approaches and strategies to address tobacco use. However, in light of competing priorities for limited resources, health plans may be challenged to sustain the improvements they have made from 1997 to 2002. Cost modeling and the development of a business case model for smoking cessation may hold promise by assisting some plans to leverage the body of literature that supports the cost-effectiveness of tobacco cessation treatment (6,20-23).

Just as challenges lay ahead, so do many important and potentially exciting opportunities. Health plans are in a key position to implement operational policies and programs that can reduce the prevalence of tobacco use and positively impact the health of millions of individuals. Health plans have the opportunity to sustain and expand access to tobacco cessation treatments and services such as pharmacotherapies and counseling services. As new evidence emerges, health plans have the flexibility to model new tobacco cessation benefits and promote them widely to their membership. They also have the opportunity to influence large purchasers of health care services by communicating the value of tobacco cessation services and expanding their field of influence from the clinical and provider setting to the broader community. By participating in community-wide campaigns and policy initiatives that support tobacco cessation and prevention, stakeholders can influence and help control tobacco use.

In summary, the results of the 2002 ATMC survey reflect both tremendous accomplishments and important opportunities for health plans to collaborate in tobacco control efforts. With appropriate support, analytical tools, and resources it is likely that health plans, clinicians, providers, and consumers will continue to evolve in their efforts to reduce the negative consequences of tobacco use.

## Figures and Tables

**Table 1:** Results from the 2002 Addressing Tobacco in Managed Care Survey (N = 152), United States

	**% Yes**
Plan has written clinical guidelines for smoking cessation	71.1
Plan uses internally developed clinical guidelines for smoking cessation	56.6
Plan uses the 2000 U.S. Public Health Service Clinical Practice Guideline	5.3
Plan uses the 1996 Agency for Health Care Policy and Research Guideline	3.3
Plan uses guidelines from some other source	5.9
Plan is able to identify individual members who smoke	71.7

**Data sources used by plans to identify individual members who smoke (among plans that can identify smokers):**	

Health risk appraisal	89.9
Telephone survey	74.1
Sample of medical records	60.6
Administrative data review	53.2
Mail-based survey	48.6
Electronic medical record	48.6
Enrollment information	6.4

**Plan provides full coverage for:**	

Bupropion (as Wellbutrin)	79.2
Bupropion (as Zyban)	41.1
Prescription NRT^a^ nasal spray	35.8
Prescription NRT inhaler	35.8
NRT over-the-counter patches	8.6
NRT over-the-counter gum	4.6

**Plan provides full coverage for:**	

Telephone counseling	51.7
Face-to-face counseling	41.1
Self-help materials (e.g., booklets, videos, audiotapes, tailored mailings)	25.8
Individual counseling of pregnant women	19.2
Group counseling or classes	15.9
Plan has annual or lifetime limits on coverage for smoking cessation interventions	15.1
Plan allows patients to self-refer to smoking cessation services	59.3

**Plan requires providers to:**	

Ask new patients about their smoking status	61.2
Include smoking status as a vital sign (i.e., ask about and document status at every visit)	54.3
Strongly advise all patients who smoke to quit	44.1
Refer smokers to intensive treatment as appropriate	33.6
Arrange for follow-up with patients trying to quit smoking	30.3

**Plan has specific strategy to address smoking cessation during:**	

Pregnancy	56.6
Treatment for other chronic illness	52.0
Post-myocardial infarction	46.7
Postpartum visits (relapse prevention)	46.7
Adolescence	28.9
Pediatric visits (secondhand smoke)	28.3
Hospitalization	7.2

**Plan has guidelines, protocols, or pathways to address smoking cessation during:**	

Pregnancy	65.1
Treatment for other chronic illness	61.8
Post-myocardial infarction	57.2
Adolescence	57.2
Pediatric visits (secondhand smoke)	55.3
Postpartum visits (relapse prevention)	53.3
Hospitalization	36.2
Plan funds a full- or part-time tobacco control program staff position	19.1

**Plan used the following strategies with providers and/or their office staff in the past year to promote smoking cessation:**	

Provider education	69.8
Providing prompts and reminders to encourage providers to address tobacco control	53.2
Elimination of pre-authorization requirements for smoking cessation interventions	40.1
Increased reimbursement for smoking cessation counseling/assistance	34.2
Incentives for providers and their staff to effectively address tobacco	4.6
Increased amount of time that providers can spend with patients	2.0

**Barriers limiting plan’s ability to address tobacco control:**	

Resource barriers (e.g., staff, funding, competing priorities)	73.5
System barriers (e.g., poor data collection, reporting, record maintenance)	40.7
Lack of patient demand	39.7
Lack of purchaser demand	38.4
Delayed economic return on investment	33.1

a NRT indicates nicotine replacement therapy.

**Table 2:** Tobacco Control Activities by Size of Health Plan: 2002 Addressing Tobacco in Managed Care Survey (N = 152), United States

	**⩽250,000 Members** **(N = 102)** **% Yes**	**>250,000 Members** **(N = 50)** **% Yes**	***P* a**
Plan has a written clinical guideline for smoking cessation	62.4	90.0	**<.001**

**Plan provides full coverage for:**			

NRT^b^ over-the-counter gum	3.0	8.0	.17
NRT over-the-counter patches	7.9	10.0	.67
NRT inhaler	42.6	22.0	**.01**
NRT nasal spray	42.6	22.0	**.01**
Bupropion (as Zyban)	47.5	28.0	**.02**
Bupropion (as Wellbutrin)	80.0	77.6	.73

**Plan provides full coverage for:**			

Telephone counseling	62.4	30.0	**<.001**
Face-to-face counseling	52.5	18.0	**<.001**
Group counseling or classes	14.9	18.0	.62
Individual counseling of pregnant women	14.9	28.0	.054
Self-help materials	20.8	36.0	**.04**
Plan has annual or lifetime limits on coverage for smoking cessation interventions	17.0	28.6	.10
Plan allows patients to self-refer to smoking cessation services	68.3	40.8	**.001**

**Plan requires providers to:**			

Ask new patients about smoking status	48.0	88.0	**<.001**
Include smoking status as a vital sign (i.e., ask about and document status at every visit)	39.2	85.7	**<.001**
Strongly advise all patients who smoke to quit	46.1	83.3	**.001**
Refer smokers to intensive treatment as appropriate	37.3	26.0	.17
Arrange for follow-up with patients trying to quit smoking	35.3	20.0	.054
Plan able to identify individual members who smoke	90.2	34.0	**<.001**

**Plan has a specific strategy to address smoking cessation during:**			

Adolescence	5.9	76.0	**<.001**
Pregnancy	42.2	86.0	**<.001**
Postpartum visits (relapse prevention)	33.3	74.0	**<.001**
Pediatric visits (secondhand smoke)	5.9	74.0	**<.001**
Post-myocardial infarction	33.3	74.0	**<.001**
Treatment for other chronic illness	39.2	78.0	**<.001**
Hospitalization	3.9	14.0	**.02**

**Plan has guidelines, protocols, or pathways to address smoking cessation during:**			

Adolescence	52.0	68.0	.06
Pregnancy	56.9	82.0	**.002**
Postpartum visits (relapse prevention)	46.1	68.0	**.01**
Pediatric visits (secondhand smoke)	48.0	70.0	**.01**
Post-myocardial infarction	50.0	72.0	**.01**
Treatment for other chronic illness	53.9	78.0	**.004**
Hospitalization	48.0	12.0	**<.001**
Plan funds a tobacco control program staff position	14.7	28.0	**.05**

a **Boldface** indicates a significant difference.

b NRT indicates nicotine replacement therapy.

**Table 3:** Comparison of Data from the 1997, 2000, and 2002 Addressing Tobacco in Managed Care Surveys, United States

	**1997** **(N = 323)** **(% Yes)**	**2000** **(N = 85)** **(% Yes)**	**2002** **(N = 152)** **(% Yes)**	***P*** a

**Plan provides full coverage for:**				

Any pharmacotherapy for smoking cessation	25.0	59.2	88.8	**<.001**
Zyban	17.6	37.2	41.1	.57
Any over-the-counter NRT^b^	6.6	14.9	8.6	**.004**
NRT only with program enrollment	25.0	26.0	10.8	**.004**

**Plan provides full coverage for:**				

Telephone counseling	32.8	36.8	51.7	**.04**
Face-to-face counseling	26.6	23.6	41.1	**.01**
Group counseling or classes	35.7	37.0	15.9	**<.001**
Self-help materials	54.1	56.6	25.8	**<.001**
Plan provides full coverage for any behavioral or pharmacotherapy	75.0	94.4	98.0	.28

**Plan requires providers to:**				

Ask new patients about smoking status	NA^c^	74.1	61.2	**.02**
Include smoking status as a vital sign (i.e., ask about and document status at every visit)	NA	43.5	54.3	.28
Strongly advise all patients who smoke to quit	NA	68.3	44.1	**.02**
Refer smokers to intensive treatment as appropriate	NA	24.7	33.6	.33
Arrange for follow-up with patients trying to quit smoking	NA	36.5	30.3	.15
Plan able to identify individual members who smoke	14.9	27.1	71.7	**<.001**

**Plan has a specific strategy to address smoking cessation during:**				

Adolescence	17.6	24.2	28.9	.46
Pregnancy	45.0	59.0	56.6	.72
Postpartum visits (relapse prevention)	13.6	30.5	46.7	**.02**
Pediatric visits (secondhand smoke)	15.8	17.3	28.3	.06
Post-myocardial infarction	21.7	27.2	46.7	**.004**
Treatment for chronic illness	22.6	31.3	52.0	**.002**
Plan funds a full- or part-time tobacco control program staff position	7.7	23.5	19.1	.15

a **Boldface** indicates a significant difference.

b NRT indicates nicotine replacement therapy.

c NA indicates data not available because question was not included in 1997 ATMC survey.
